# The Impact of Histological Variants on Oncological Outcomes in Patients with Muscle Invasive Bladder Cancer Treated with Radical Cystectomy

**DOI:** 10.5152/tud.2023.22223

**Published:** 2023-07-01

**Authors:** Özgür Efiloğlu, Mehmet Çağlar Çakıcı, Gözde Kır, Ayberk İplikçi, Turgay Turan, Gözde Ecem Cecikoğlu, Asıf Yıldırım

**Affiliations:** 1 Department of Urology, Istanbul Medeniyet University, Istanbul, Turkey; 2 Department of Pathology, Istanbul Medeniyet University, Istanbul, Turkey

**Keywords:** Cystectomy, histology, neoplasms, urinary bladder

## Abstract

**Objective:**

Bladder cancer is a heterogeneous entity characterized by a wide range of different morphologies. The aim of this study was to investigate the prognostic effect of bladder tumor with variant histology that is treated with radical cystectomy on oncological outcomes.

**Methods:**

One hundred eighty-six patients who underwent radical cystectomy between September 2001 and June 2020 were included in the study. The patients were divided into 2 groups variant histology group (n = 54) and transitional cell cancer group (n = 132). Clinicopathologic data were compared between the two groups.

**Results:**

The groups were similar in terms of demographic characteristics. In the multivariate analysis of cancer-specific survival in transitional cell cancer against variant histology, high-grade detection of primary transurethral bladder tumor pathology, cystectomy pT, cystectomy positive lymph node, and positive surgical margin in cystectomy were determined to be statistically significant. Diagnosis of pT2 and high grade of primary transurethral bladder tumor pathology, cystectomy ≥ pT3, cystectomy positive lymph node, and positive surgical margin in cystectomy were statistically significant in multivariate analysis of overall survival. Cancer-specific survival time was estimated at 65.1 ± 8.3 months for variant histology and 134.2 ± 10.4 months for transitional cell cancer (*P* = .004). The estimated overall survival time was 61.9 ± 8.0 months in variant histology and 119.0 ± 9.8 months in transitional cell cancer (*P* = .014).

**Conclusion:**

Pathological features and prognosis of bladder cancer with variant histologies are worse than those of pure urothelial bladder cancer. Overall survival and cancer-specific survival are shorter in bladder cancer with variant histology than in pure urothelial bladder cancer. Following the diagnosis of variant histology in transurethral bladder tumor, poor prognosis must be considered in the treatment plan.

**Main Points**

- This study investigated the prognostic impact of bladder tumors with variant histology (VH) treated with radical cystectomy (RC) on oncologic outcomes.
- In this study, it was found that patients with VH had worse cancer-specific survival (CSS) and overall survival (OS) compared with patients with transitional cell cancer. In univariate analysis, VH was found to be predictive of patients’ CSS and OS at cystectomy.
- After the diagnosis of VH in transurethral bladder tumor, the poor prognosis should be considered in the treatment plan.

## Introduction

Bladder cancer is the tenth most frequently diagnosed cancer worldwide, with approximately 573 000 new cases and 213 000 deaths in 2020. It is approximately 4 times more common in men, with incidence and mortality rates of 9.5 and 3.3 per 100 000 in men.^1^

**Supplementary Figure 1.**
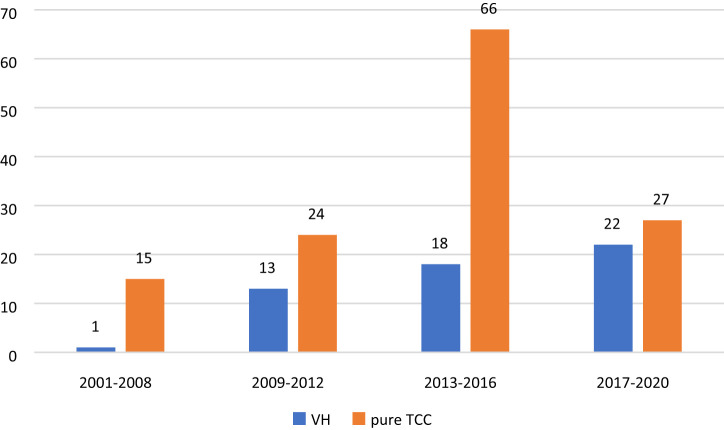
The rate of VH presence in cystectomy material according to years.

Recognition of variant histology (VH) in bladder cancer has recently increased due to advances in immunohistochemical techniques and understanding of the effects of variant histologies on patients’ prognostic outcomes.^2^ Although the frequency of VH is uncertain, previous studies have reported a wide range of VH prevalence from 7% to 81%.^3^ This wide range may be due to the lack of standardized reporting methodologies for transitional cell cancer (TCC).^4^ In our study, the VH sequence continues over the years in agreement with the literature (Supplementary Figure 1).

Various urology and oncology associations, such as the European Association of Urology (EAU) and the European Association of Medical Oncology (ESMO), publish clinical practice guidelines based on the latest evidence and expert opinions. However, in some areas of variant bladder cancer management, the evidence is limited and/or conflicting. The pathological features and prognosis of bladder cancer with different histologies are different from pure urothelial bladder cancer. Evidence for response to systemic therapy in variant histologies is scarce and variable.^5^

The aim of this study was to investigate the prognostic effect of bladder tumor with VH that has been followed up regularly from the date of initial diagnosis and treated with radical cystectomy (RC) on oncological outcomes.

## Material and Methods

The ethics committee approval of the Istanbul Medeniyet University was obtained (2020/0539) prior to the initiation of the study. An electronic data search was carried out on prospectively kept patient files dated between September 2001 and June 2020. Among 1946 patients who were followed up for bladder cancer in our hospital, 224 patients with TCC who underwent RC were identified. The data of 186 patients with complete followed ups since the first transurethral bladder tumor (TUR-B) surgery were included in the study. Informed consent was obtained from all individual participants included in the study. Patients with nephroureterectomy due to upper urinary tract carcinoma, patients who underwent salvage cystectomy, and patients with distant metastases before cystectomy were excluded from the study. Prior to RC, patients were evaluated with whole-body computed tomography (CT) scanning or magnetic resonance imaging (MRI) to detect distant metastases. Neoadjuvant, adjuvant chemotherapy, and radiotherapy were administered according to the tumor stage, the general health status of the patients, and the patients’ preferences.

The patients’ post-TUR-B follow-up was performed according to the current EUA guideline of that period. After RC, patients were followed up for 2 years with laboratory tests, physical examination, CT scans, radiological imaging, and urine cytology. Additional radiographic evaluations (bone scans, MRI, positron emission tomography–computed tomography (PET-CT), etc.) were performed at the discretion of the treating physician when clinically indicated. After the second year, the same follow-up protocol was done every 6 months.

We compared histopathological diagnoses of variants of invasive urothelial carcinoma in the TUR-B and RC specimens. Transurethral bladder tumor and radical cystectomy specimens were processed according to standard pathological procedures. The hematoxylin and eosin (H&E)-stained sections of TUR-B specimens of all patients were evaluated independently by 2 genitourinary pathologists. Each case was re-classified according to the 2016 WHO/ International Society of Urologic Pathology classification. Squamous differentiation, glandular differentiation, nested, micropapillary, lymphoepithelioma-like, sarcomatoid, giant cell, clear cell, lipid cell, plasmacytoid, and undifferentiated morphology were assessed as VH. Percentages of variant components were not included in the analysis due to a lack of standard reporting.

### Statistical Analysis

For descriptive statistics in the study, quantitative variables with normal distribution were presented as mean ± standard deviation and variables without normal distribution were presented as median (range). The normality of the data was analyzed using the Kolmogorov–Smirnov test. The *t*-test was used for the variables of quantitative data that had a normal distribution, and the Mann–Whitney *U* test was utilized for the others. Comparison of categorical variables was performed by Pearson chi-square test and Fisher’s exact test. Cumulative survival rates were calculated using the Kaplan–Meier method, and the significance of differences in the survival rate was analyzed using the log-rank test. Univariate and multivariate Cox proportional hazards regression were performed to determine the factors that were associated with cancer-specific survival (CSS) and overall survival (OS). Likelihood of a type I error was considered *α* = 0.05 for all tests. Statistical analyses were performed using Statistical Package for Social Sciences version 20.0 (IBM SPSS Crop.; Armonk, NY, USA) packaged software program.

## Results

Of the 186 patients, 166 (89.2%) were male and 20 (10.8%) were female. The mean follow-up time was 56.4 ± 49.3 months. Groups were similar in terms of demographic characteristics (Table 1).

When the first pathologies by which the patients were diagnosed with bladder tumor were examined, the 2 groups had similar characteristics in terms of pathological T (pT) stages, grade, and accompanying carcinoma in situ (CIS). In the last TUR-B pathologies before cystectomy, pT2 (79.6% vs. 60.6% *P* = .018) was higher in the VH group, but the rates of grade and accompanying CIS were similar. During cystectomy, VH was detected in 36 (66.7%) of 54 patients with variant pathology before cystectomy (Table 1).

When the cystectomy pathologies were examined, no pT0 stage was found in the VH group. In the TCC group, pT0 stage was detected in cystectomy of 24 (18.2%) patients, 4 of whom had neoadjuvant chemotherapy. In cystectomy, > pT3 stage and high grade were detected more in the VH group (*P* = .001). The number of lymph nodes removed during cystectomy, the number of positive lymph nodes, accompanying prostate cancer, and prostate cancer stages were similar in both groups. Squamous cell differentiation was seen in 22 (40.7%) of 54 patients who had VH in cystectomy and more than one variant was found in 8 (14.8%) patients. The distribution of variants is given in Table 2. There was no difference in the types of diversion applied in cystectomy (*P* = .245) and complication rates (*P* = .801) in both groups.

There was no variant pathology in TUR-B performed prior to cystectomy in 18 patients with variant pathology detected in RC. The distribution of cystectomy pathologies of these patients was squamous cell differentiation in 4 (22%) patients, nested and micropapillary variant in 3 (17%) patients, glandular cell differentiation in 2 (11%) patients, and lymphoepithelioma-like, sarcomatoid, clear-cell, plasmacytoid, undifferantial, and multiple variants were detected in 1 (5%) patient each.

When TUR-B pathologies of 22 patients with squamous cell differentiation variant pathology in cystectomy were examined, no variant was detected in 4 patients. Glandular cell differentiation and micropapillary variant were detected in 1 patient each. Pathologies of 29 out of 54 patients with variant pathology in cystectomy were found to be compatible with TUR-B pathology.

High grade of primary TUR-B pathology, cystectomy pT, cystectomy positive lymph node, and positive surgical margin in cystectomy were found to be both statistically significant on both univariable and multivariable analyses of CSS against histological variants in TCC (Table 3).

Table 4 shows the multivariate model created with significant values in univariate analysis of OS in TCC against histological variants. Age, pT2 and high grade of primary TUR-B pathology, cystectomy ≥ pT3, cystectomy positive lymph node, and positive surgical margin in cystectomy were found to be statistically significant.

**Figure 1.**
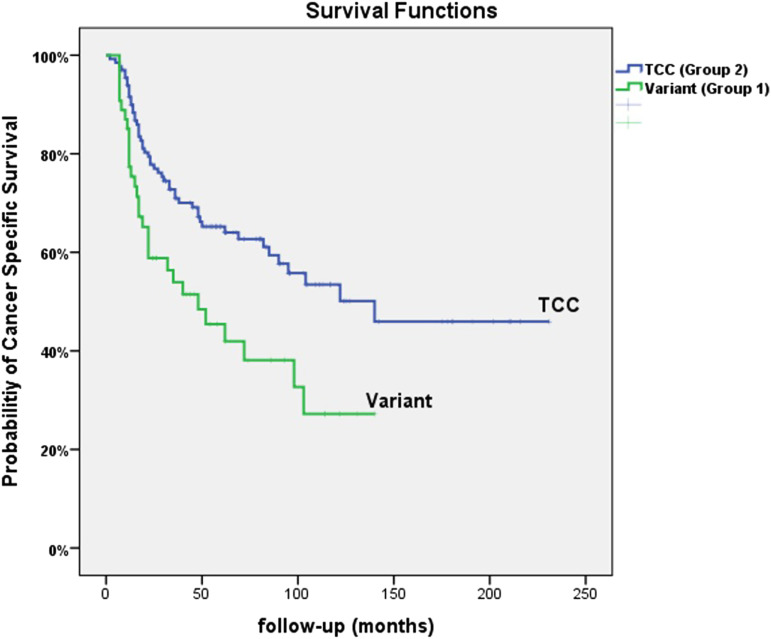
Kaplan–Meier curve for cancer-specific survival. The *P* value of the log-rank method was .004 and the chi-square value was 8.438. The estimated cancer-free survival time was 65.1 ± 8.3 months in group 1 and 134.2 ±10.4 months in group 2 (*P* = .004).

**Figure 2.**
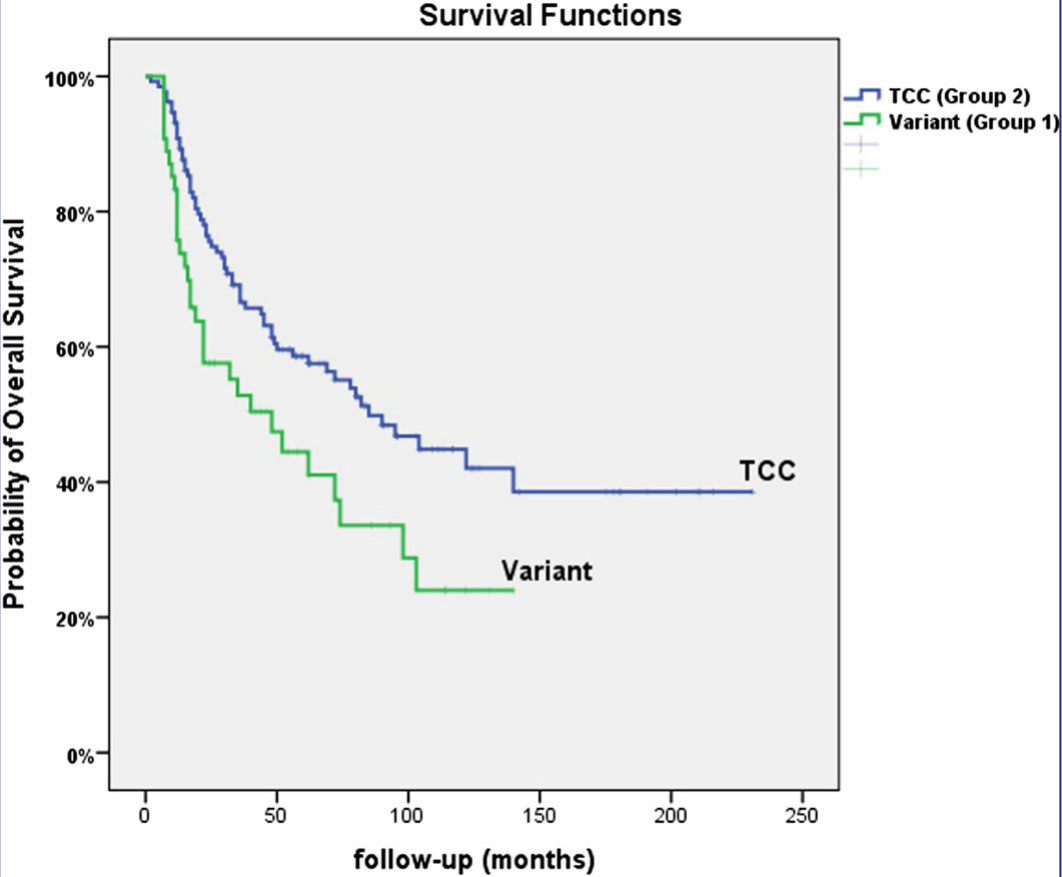
Kaplan–Meier curve for overall survival. The *P* value of the log-rank method was .011 and the chi-square value was 6.065. The estimated cancer-free survival time was 61.9 ± 8.0 months in group 1 and 119.0 ± 9.8 months in group 2 (*P* = .014).

Figure 1 shows Kaplan–Meier plots for predictions of cancer-free survival in TCC versus histological variants. The cancer-free survival time was estimated as 65.1 ± 8.3 months for VH and 134.2 ± 10.4 months for TCC (*P* = .004). The estimated overall survival (OS) time was 61.9 ± 8.0 months in VH and 119.0 ± 9.8 months in TCC (*P* = .014) (Figure 2).

The rate of patients receiving adjuvant chemotherapy was higher in the VH group. When the factors affecting survival were examined, the presence of VH and the use of adjuvant CT were effective in both CSS (*P* = .001) and OS (*P* = .001) in univariate analysis, but their effect was not statistically significant in multivariate analysis.

## Discussion

In this study, it was observed that patients with VH had worse CSS and OS compared to patients with pure TCC. On univariate analyses, VH pathology at cystectomy was found to predict poorer CSS and OS; however, this was not statistically significant on multivariate analyses after adjusting for Charlson comorbidity index and pT stages. Similar to our study, 2 multicenter studies with a high number of patients found VH as predictive in CSS in univariate analysis but ineffective in multivariate analysis.^6,7^

There are studies showing that VH is associated with poorer survival on multivariate analysis. Nadeem et al^8^ evaluated 201 patients that underwent RC and showed that age, gender distribution, T stage, and nodal status were not significant in predicting survival in multivariate analysis, but variant pathology was an independent risk factor for CSS. Variant pathology has also been shown to have a significant effect on OS in Cox regression analysis (hazard ratio (HR), 2.81; 95% confidence interval (CI), 1.32-5.97). However, the number of variants in that study was limited to 19 patients.^8^ In Böyük et al’s study of 223 patients undergoing radical cystectomy, pathologic stage of disease, and presence of VH were found to be associated with lower CSS in the multivariate model.^9^ In a meta-analysis of 23 studies involving 22 072 patients, VH was significantly associated with worse CSS (pooled HR 1.37, 95% CI).^10^ On the other hand, Takemoto et al^11^ found that VH was not effective on OS in both univariate and multivariate analyses in a series of 102 patients who underwent RC. The reason for the different oncological results in the studies can be explained by the heterogeneity of variant subgroups in the studies, varying histologies, varying cohort sizes, heterogeneous study populations, and specific differences in reporting methodologies for differences in treatment modalities. In a systematic review of 33 articles and 19 702 patients, advanced age, high tumor grade, lymph node metastasis, lymphovascular invasion, and positive surgical margin statistically significantly predicted CSS according to RC (*P* ≤ .05). However, it has been reported that gender, CIS, VH, and adjuvant chemotherapy may not be associated with CSS. This systematic review has some limitations such as most being retrospective cohort studies, and some studies having significant heterogeneity.^12^

There are studies stating that VH subgroups also differ in terms of survival among themselves. Monn et al^13^ conducted subgroup analyses and determined the micropapillary variant and plasmasoid variant as independent risk factors that doubled the risk of death from all causes in multivariate analysis. However, they did not detect any difference in the risk of death associated with squamous differentiation or the sarcomatoid variant.^13^ Unlike this study, Naspro et al^14^ found that only sarcomatoid, squamous differentiation, and small cell variant were associated with cancer-specific mortality in multivariate analysis. Moreover, they reported that none of the variant subtypes had an effect on overall mortality in multivariate analysis.^14^ In our study, survival analysis of subgroups of variant pathologies could not be performed due to insufficient numbers.

Standard therapy for patients with urothelial muscle-invasive bladder cancer (MIBC) and MIBC with VH is RC. Combination chemotherapies containing neoadjuvant cisplatin improve OS by 5%-8% in 5 years.^15^ However, there is insufficient evidence that this benefit of neoadjuvant therapy is also valid in VH. In a retrospective study by Vetterlein et al,^3^ patients with neuroendocrine tumors were found to have better OS rates and lower non-organ-restricted disease rates during radical cystectomy. Neoadjuvant chemotherapy for micropapillary, sarcomatoid variant, or adenocarcinoma decreased non-organ-specific disease rates but did not affect OS.^3^ Based on this study, the EAU–ESMO consensus recommends that small cell neuroendocrine variant bladder urothelial carcinoma be treated with neoadjuvant chemotherapy followed by local therapy.^5^ In our study, neoadjuvant therapy was applied at a similar rate in both groups. Univariate and multivariate analyses showed no effect on overall and disease-specific survival. Prospective randomized studies in the literature on this remain scarce.

Diagnosis of histological variants in transurethral resection of the bladder is difficult due to the small amount of tissue obtained by endoscopic resection, coagulation of the tissue, and coagulation-induced artifacts. Inaccurate diagnosis of VH affects risk stratification, prognosis, and treatment decisions, especially when treatment may differ based on VH.^16^ Pathologists’ awareness of the potential impact of histologic variants may have led to more attention being paid to these entities over the years.^6^ From a technical point of view, the en bloc TUR should be evaluated in the context of the diagnosis of VH.^17^ There are studies reporting the accurate and conflicting results of TUR-B in the evaluation of the presence and type of VH. Moschini et al^17^ found a weak agreement between the findings on TUR-B and RC. Compliance depends on the type of VH. A low level of agreement was found in the micropapillary variant, while a moderate agreement was found in sarcomatoid, small cell, and squamous variants.^17^ Abufaraj et al^18^ reported 83.6% agreement between TUR-B and RC. Variant histology was detected in 11.2% of TUR-B specimens and 25.4% of RC specimens. In our study, there was a 53.7% agreement rate between TUR-B and RC. Concordance subgroup analyses of the histologic variants are shown in Supplementary Tables 1 and 2. In the current study, survival analyses were calculated not after RC but from the moment of first diagnosis, assuming that variant pathology could also be in previous TUR-Bs but could not be detected due to the reasons listed above.

Our study has some limitations. Although the data were collected prospectively, the analyses were done retrospectively. Despite being one of the largest single-center cohorts, the overall sample size of the study is limited. In addition, the limited number of patients in VH subgroups did not allow for subgroup analyses. Therefore, our results need to be confirmed by prospective, multi-center studies.

Pathological features and prognosis of bladder cancer with variant histologies are worse than those of pure urothelial bladder cancer. Overall survival (approximately 58 months) and CSS (approximately 69 months) are shorter in bladder cancer with VH than in pure urothelial bladder cancer. After the diagnosis of VH in TUR-B, the poor prognosis must be considered in the treatment plan.

## Figures and Tables

**Table 1.** Patients’ Demographic Characteristics and Pre-Cystectomy Oncological Features

	Group 1 (n = 54)	Group 2 (n = 132)	*P*
Age (years), mean ± SD	66.2 ± 9.0	65.9 ± 9.2	.854
Gender, n (%) Male Female	45 (83.3) 9 (16.7)	121 (91.7) 11 (8.3)	.096
Body mass index, kg/m^2^; mean ± SD	27.6 ± 3.7	27.2 ± 4.4	.526
Charlson comorbidity index, mean ± SD	6.5 ± 2.5	5.7 ± 2.0	.025
Family history, n (%)	1 (1.9)	5 (3.8)	.674^*^
Smoking (pack-year)	33.1 ± 25.9	35.0 ± 22.7	.637
Primary pT stage, n (%) pTa pT1 pT2	5 (9.3) 19 (35.2) 30 (55.6)	14 (10.6) 61 (46.2) 57 (43.2)	.300
Primary grade, n (%) Low grade High grade	3 (5.6) 51 (94.4)	17 (12.9) 115 (87.1)	.143
Concomitant CIS, n (%)	7 (13)	10 (7.6)	.247
Pre-cystectomy pT stage, n (%) pTa pT1 pT2	4 (7.4) 7 (13) 43 (79.6)	8 (6.1) 44 (33.3) 80 (60.6)	.018
Pre-cystectomy grade, n (%) Low grade High grade	1 (1.9) 53 (98.1)	10 (7.6) 122 (92.4)	.268^*^
Pre-cystectomy CIS, n (%)	11 (20.4)	14 (10.6)	.076
Pre-cystectomy variant histology, n (%) Squamous cell differentiation Glandular cell differentiation Micropapillary Sarcomatoid Multiple	36 (66.7) 17 (31.5) 2 (3.7) 5 (9.3) 6 (11.1) 6 (11.1)	-	

*Fisher’s Exact test. CIS, carcinoma in situ; pT, pathologic T in TNM classification.

**Table 2.** Patients’ Cystectomy Oncological Features and Perioperative Outcomes

	Group 1 (n = 54)	Group 2 (n = 132)	*P*
Time to cystectomy, day	74 (10-419) 94.0 ± 74.5	62.5 (12-605) 82.0 ± 72.4	.134^†^
Cystectomy pT stage, n (%) pT0 pTis pTa pT1 pT2 pT3 pT4	——— 3 (5.6) 14 (25.9) 25 (46.3) 12 (22.2)	24 (18.2) 1 (0.8) 4 (3.0) 16 (12.1) 38 (28.8) 38 (28.8) 11 (8.3)	.001^*^
Cystectomy ≥pT3 stage, n (%)	37 (68.5)	49 (37.1)	<.001
Cystectomy pN stage, n (%) pN0 pN1 pN2	35 (64.8) 10 (18.5) 9 (16.7)	102 (77.3) 15 (11.4) 15 (11.4)	.212
Cystectomy grade, n (%) Low grade High grade	— 54 (100)	32 (24.2) 100 (75.8)	<.001
LN number	12.2 ± 7.0	10.7 ± 5.6	.139
Positive LN number	1.4 ± 3.4	0.7 ± 1.7	.132
Positive surgical margin, n (%)	7 (13)	7 (5.3)	.072
Cystectomy variant histology, n (%) Squamous cell differentiation Glandular cell differentiation Nested Micropapillary Lymphoepithelioma-like Sarcomatoid Clear-cell Plasmacytoid Undifferentiated Multiple	22 (40.7) 3 (5.6) 3 (5.6) 9 (16.7) 2 (3.7) 4 (7.4) 1 (1.9) 1 (1.9) 1 (1.9) 8 (14.8)		
Concomitant prostate cancer, n (%)	9 (16.7)	23 (17.4)	.901
Prostate cancer pT stage, n (%) pT2 pT3	9 (16.7) —	21 (15.9) 2 (1.5)	.659^*^
Diversion type, n (%) Ileal diversion Orthotopic neobladder Cutaneous diversion	43 (79.6) 5 (9.3) 6 (11.1)	111 (84.1) 15 (11.4) 6 (4.5)	.245
Complication, n (%) Clavien-Dindo score 1-2 Clavien-Dindo score 3-5	24 (44.4) 6 (11.1)	60 (45.5) 16 (12.1)	.801
Neoadjuvant CT, n (%)	7 (13)	13 (9.8)	.534
Adjuvant CT, n (%)	25 (46.3)	35 (26.5)	.009
Adjuvant RT, n (%)	4 (7.4)	12 (9.1)	.710

N, Lymph node in TNM classification; LN, lymph node; pT, pathologic T in TNM classification, CT, computed tomography; RT, radiation therapy.

**Table 3.** Univariate and Multivariate Cox Regression Analysis of Factors Affecting Cancer-Specific Survival

	Univariate Model			Multivariate Model		
	HR	(95% CI)	*P *	HR	(95% CI)	*P*
Age	1.015	0.990-1.040	.239			
Gender (ref: male)	1.211	0.624-2.349	.542			
BMI	1.016	0.966-1.068	.746			
CCI	1.279	1.171-1.396	<.001			
Smoking package/year	0.992	0.982-1.001	.089			
Family history	0.608	0.149-2.477	.488			
Primary pT stage	1.519	1.071-2.154	.019			
Primary Grade (ref: low grade)	2.065	0.896-4.757	.089	3.358	1.387-8.127	.007
Concomitant CIS	1.030	0.448-2.372	.944			
pT pre-cystectomy	1.397	0.943-2.071	.096			
pT2 pre-cystectomy	1.733	1.061-2.830	.028			
Grade pre-cystectomy	1.156	0.467-2.861	.753			
CIS pre-cystectomy	1.027	0.544-1.941	.933			
Variant pre-cystectomy	1.685	1.006-2.824	.047			
Time to cystectomy	1.002	0.998-1.005	.400			
>60 days	1.094	0.706-1.694	.689			
>90 days	1.485	0.934-2.361	.094			
pT cystectomy	1.621	1.374-1.912	<.001			
pT3-4 cystectomy	4.652	2.851-7.590	<.001	3.364	1.990-5.686	<.001
pN cystectomy	2.428	1.864-3.162	<.001			
pN positivity cystectomy	4.208	2.691-6.580	<.001	2.623	1.599-4.301	<.001
Grade cystectomy	7.493	2.360-23.792	.001			
PSM	4.087	2.172-7.690	<.001	3.670	1.849-7.285	<.001
LN number	0.996	0.959-1.034	.828			
Positive LN number	1.176	1.115-1.239	<.001			
Variant histology cystectomy	1.927	1.224-3.033	.005			
Concomitant PCa	1.156	0.658-2.029	.615			
Diversion type	1.398	0.979-1.996	.065			
Clavien-Dindo score	1.013	0.839-1.223	.892			
Neoadjuvan CT	1.436	0.713-2.890	.311			
Adjuvan CT	2.674	1.719-4.160	<.001			
RT	2.030	1.140-3.617	.016			

*The *P *value of the model was <.001 and the chi-square value was 89.313.

BMI, Body mass index; 95%CI, confidence interval; CCI, Charlson comorbidity index; CIS, carcinoma in situ; CT, chemotherapy; LN, lymph node; N, lymph node in TNM classification; PCa, prostate cancer; PSM, positive surgical margin; pT, pathologic T in TNM classification; pN, pathologic N in TNM classification; RT, radiation therapy.

**Table 4.** Univariate and Multivariate Cox Regression Analysis of Factors Affecting Overall Survival

	Univariate Model			Multivariate Model		
	HR	(95% CI)	*P *	HR	(95% CI)	*P*
Age	1.024	1.001-1.047	.042	1.032	1.009-1.057	.007
CCI	1.244	1.146-1.351	<.001			
Smoking package/year	0.996	0.988-1.005	.386			
Family history	0.768	0.243-2.428	.653			
Primary pT stage (ref: pTa)	1.600	1.156-2.215	.005			
Primary pT2	1.902	1.266-2.857	.002	1.755	1.129-2.730	.013
Primary Grade (ref: low grade)	2.109	0.974-4.564	.058	2.464	1.062-5.716	.036
Concomitant CIS	1.037	0.479-2.243	.927			
pT pre-cystectomy (Ref: pTa)	1.511	1.041-2.193	.030			
pT2 pre-cystectomy	1.840	1.164-2.907	.009			
Grade pre-cystectomy	1.394	0.566-3.435	.470			
CIS pre-cystectomy	0.939	0.513-1.721	.839			
Variant pre-cystectomy	1.588	0.976-2.584	.063			
Time to cystectomy	1.002	0.999-1.005	.163			
>60 days	1.077	0.719-1.612	.719			
>90 days	1.463	0.951-2.250	.083			
pT cystectomy (Ref: pT0)	1.459	1.254-1.699	<.001			
pT3-4 cystectomy	3.414	2.229-5.228	<.001	2.344	1.469-3.741	<.001
pN cystectomy	2.141	1.662-2.758	<.001			
pN positivity cystectomy	3.244	2.135-4.930	<.001	2.472	1.535-3.983	<.001
Grade cystectomy	4.432	1.933-10.158	<.001			
PSM	3.591	1.924-6.701	<.001	3.833	1.948-7.542	<.001
LN number	0.990	0.956-1.025	.559			
Positive LN number	1.162	1.101-1.226	<.001			
Variant histology cystectomy	1.696	1.105-2.601	.016			
Concomitant PCa	1.372	0.836-2.253	.211			
Diversion type	1.229	0.862-1.751	.255			
mClavien score	1.055	0.888-1.254	.540			
Neoadjuvant CT	1.396	0.721-2.705	.322			
Adjuvant CT	2.019	1.338-3.045	.001			
RT	1.662	0.941-2.934	.080			

^*^The *P *value of the model was <.001 and the chi-square value was 81.834.

CCI, Charlson comorbidity index; CIS, carcinoma in situ; CT, chemotherapy; LN, lymph node; PCa, prostate cancer; PSM, positive surgical margin; pT, pathologic T in TNM classification; RT, radiation therapy.

**Supplementary Table 1.** Concordance subgroup analyses of variant histology; demographic characteristics, pre-cystectomy oncological features (Group 1: concordant in TURB/RC; Group 2: inconcordant in TURB/RC)

	Group 1 (n = 29)	Group 2 (n = 25)	*P *value
Age (years), mean ± SD	67.72 ± 8.4	64.4 ± 9.3	0.183
Gender, n (%) Male Female	22 (75.9) 7 (24.1)	23 (92) 2 (8)	0.153
Body mass index, kg/m^2^; mean ± SD	27.5 ± 3.9	27.5 ± 3.5	0.964
Charlson comorbidity index, mean ± SD	5.8 ± 2.2	7.3 ± 2.5	0.026
Family history, n (%)	3 (8)	2 (10.3)	0.387
Smoking (pack-year)	26.3 ± 24.4	40.8 ± 25.8	0.040
Primary pT stage, n (%) pTa pT1 pT2	3 (10.3) 10 (34.5) 16 (55.2)	2 (8) 9 (36) 14 (56)	0.956
Primary grade, n (%) Low grade High grade	1 (3.4) 28 (96.6)	2 (8) 23 (92)	0.591
Concomitant CIS, n (%)	4 (13.8)	3 (12)	0.845
Pre-cystectomy pT stage, n (%) pTa pT1 pT2	1 (3.4) 4 (13.8) 24 (82.8)	3 (12) 3 (12) 19 (76)	0.488
Pre-cystectomy grade, n (%) Low grade High grade	0 (0) 29 (100)	1 (4) 24 (96)	0.463
Pre-cystectomy CIS, n (%)	5 (17.2)	6 (24)	0.539
Pre-cystectomy variant histology, n (%) Squamous cell differentiation Glandular cell differentiation Micropapillary Sarcomatoid Multiple	17 (58.6) 1 (3.4) 4 (13.8) 4 (13.8) 3 (10.3)	- 1 (4) 1 (4) 2 (8) 3 (12)	

HR: hazard ratio, 95%CI: confidence interval, pT: pathologic T in TNM classification, N: lymph node in TNM classification, CIS: carcinoma in situ

**Supplementary Table 2.** Concordance subgroup analyses of variant histology; oncological features and perioperative outcomes (Group 1: concordant in TURB/RC; Group 2: inconcordant in TURB/RC)

	Group 1 (n = 29)	Group 2 (n = 25)	*P* value
Cystectomy pT stage, n (%) pT1 pT2 pT3 pT4	1 (3.4) 7 (24.1) 17 (58.6) 4 (13.8)	2 (8) 7 (28) 8 (32) 8 (32)	0.2
Cystectomy pN stage, n (%) pN0 pN1 pN2	23 (79.3) 4 (13.8) 2 (6.9)	12 (48) 6 (24) 7 (28)	0.041
Positive surgical margin, n (%)	2 (6.9)	5 (20)	0.229
Cystectomy variant histology, n (%) Squamous cell differentiation Glandular cell differentiation Nested Micropapillary Lymphoepithelioma-like Sarcomatoid Clear-cell Plasmacytoid Undifferentiated Multiple	16 (55.2) 1 (3.4) 0 (0) 4 (13.8) 0 (0) 3 (10.3) 0 (0) 0 (0) 0 (0) 5 (17.2)	6 (24) 2 (8) 3 (12) 5 (20) 2 (8) 1 (4) 1 (4) 1 (4) 1 (4) 3 (12)	
Concomitant Prostate cancer, n (%)	7 (24.1)	2 (8)	0.153
Prostate cancer pT stage, n (%) pT2	7 (24.1)	2 (8)	0.113
Diversion type, n (%) Ileal diversion Orthotopic neobladder Cutaneous diversion	25 (86.2) 0 (0) 4 (13.8)	18 (72) 5 (20) 2 (8)	0.644
Complication, n (%) Clavien-Dindo score 1-2 Clavien-Dindo score 3-5	14 (48.2) 4 (13.8)	10 (40) 2 (8)	0.499
Neoadjuvant CT, n (%)	3 (10.3)	4 (16)	0.692
Adjuvant CT, n (%)	12 (41.4)	13 (52)	0.585
Adjuvant RT, n (%)	2 (6.9)	2 (8)	0.877

HR: hazard ratio, 95%CI: confidence interval, pT: pathologic T in TNM classification, N: lymph node in TNM classification, LN: Lymph Node
